# Qualitative Approaches Towards Useful Photocatalytic Materials

**DOI:** 10.3389/fchem.2020.00817

**Published:** 2020-09-11

**Authors:** Raul Quesada-Cabrera, Ivan P. Parkin

**Affiliations:** Christopher-Ingold Laboratories, Materials Chemistry Center, Department of Chemistry, UCL (University College London), London, United Kingdom

**Keywords:** qualitative pyramid, photocatalytic properties, limitations in photocatalysis, *in situ/in operando* photocatalysis, visible-light activity, dye-sensitized processes, scalability, black TiO_2_

## Abstract

The long-standing crusade searching for efficient photocatalytic materials has resulted in a vast landscape of promising photocatalysts, as reflected by the number of reviews reported in the last decade. Virtually all of these reviews have focused on quantitative approaches aiming at developing an understanding of the underlying mechanisms behind photocatalytic behavior and the parameters that influence structure–function correlation. Less attention has been paid, however, to qualitative measures around the development and assessment of photocatalysts. These measures will contribute toward narrowing the range of potential photocatalytic materials for widespread applications. The current report provides a critical perspective over some of the main factors affecting the assessment of photocatalytic materials as a code of good practice. A case of study is also provided, where this qualitative analysis is applied to one of the most prolific materials of the last-decade, disorder-engineered, black titanium dioxide (TiO_2_).

## Introduction

**Figure 1:**
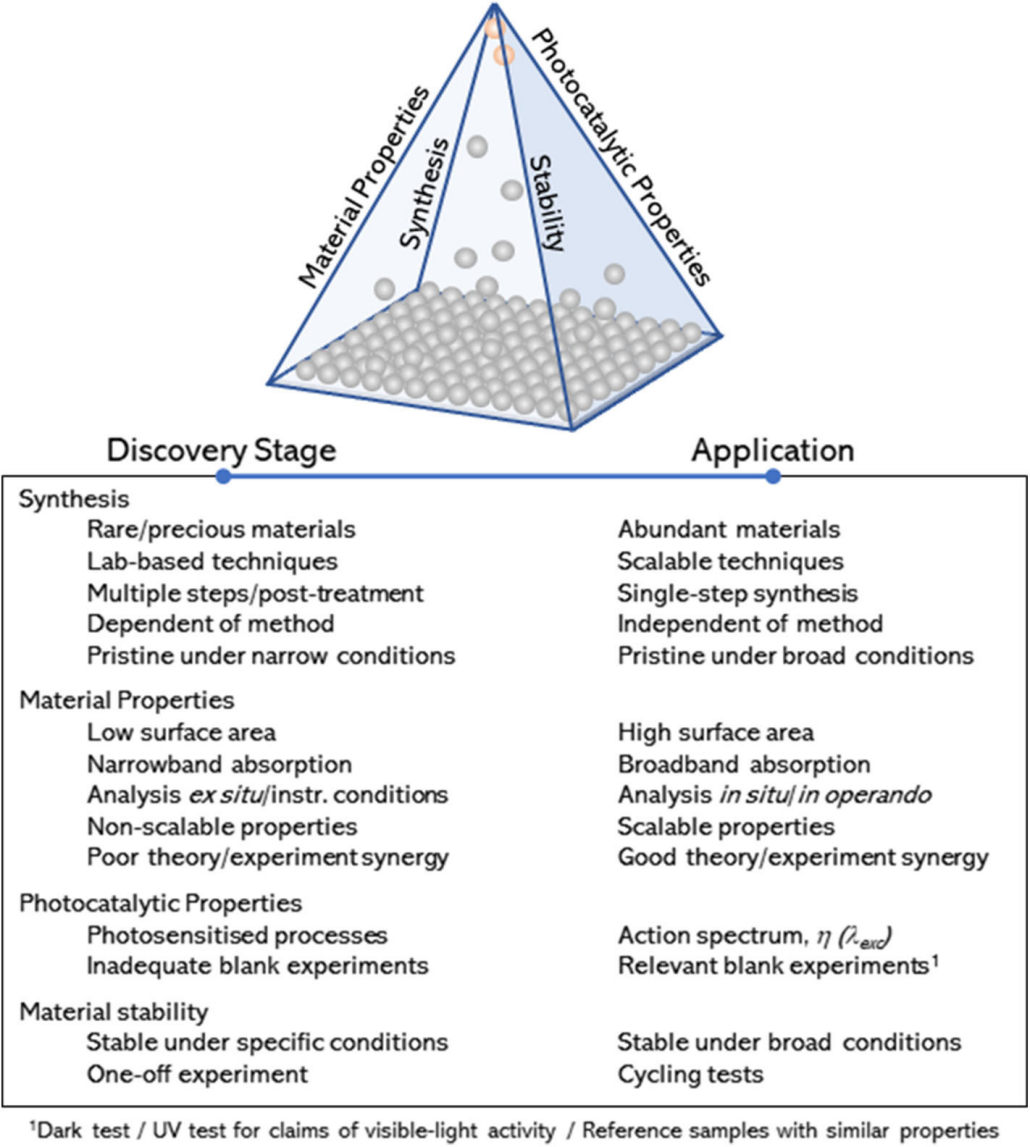
Schematic representation (*qualitative pyramid*) and description of qualitative measures considered in this review and designed to identify promising photocatalytic materials (round symbols) moving from the discovery stage to potential market applications.

The research activities in the engineering of photocatalysts has continued growing steadily over the last decades, as evidenced by the number of reviews in this subject, covering widespread experimental (Jang et al., 2012; Takanabe and Domen, 2012; Tong et al., 2012; Dozzi and Selli, 2013; Osterloh, 2013; Wang et al., 2014; Zhou et al., 2014; Li et al., 2015, 2016a, 2018; Moniz et al., 2015; Zhang et al., 2016; Bai et al., 2017, 2018; Chen et al., 2017; Takanabe, 2017; Adekoya et al., 2019; Zhao et al., 2020) and theoretical studies (Sun and Ceder, 2013; Bokarev et al., 2015; Pacchioni, 2015; Zhang and Jaroniec, 2018; Wang et al., 2019). Despite these efforts, overall efficiencies of advanced materials are still very far from industrial targets, and the global market in photocatalysis (>£1Bn) is still dominated by a single standard material—titanium dioxide (TiO_2_). So far, these efforts have rightly focused on the interplay between material properties and photocatalytic activity; i.e., they account for a *quantitative* description of the main parameters that potentially contribute to their photocatalytic performance (Takanabe, 2017). In contrast, less attention has been paid to *qualitative* measures that may allow for an assessment of (1) the preparation method and experimental factors affecting key material properties toward photocatalytic performance, (2) characterization approaches and synergy between theory and experimental work, (3) photocatalytic tests and control experiments, and (4) material stability within a broad range of experimental conditions. Attention to these qualitative approaches is widely implied but often disregarded in the literature. Drawn together over the edges of a pyramidal diagram (Figure 1), these qualitative measures can help narrowing down the range of promising photocatalysts for widespread industrial use. The list provided in Figure 1 is not exhaustive, but it points at key approaches to bring materials from the discovery stage (at the bottom of the pyramid) to practical applications (at the apex of the pyramid). It is worth noting that the diagram is not based on figures of merit denoting photocatalytic efficiency. The materials at the apex of the pyramid will be inexpensive, robust, scalable photocatalysts with proven ability toward photocatalytic behavior, consistently demonstrated under a broad range of experimental conditions. Examples of these materials are commercial standards such as TiO_2_–widely used as self-cleaning products in the market—and zinc oxide (ZnO). In contrast, promising materials, such as those based on perovskite structures, which may show outstanding efficiencies but only under restricted conditions (Snaith, 2013; Zhang et al., 2016), will remain in the middle (perhaps upper part) region of the pyramid.

This review centers the discussion around qualitative measures and the common sources of misleading conclusions in the synthesis, characterization, and assessment of photocatalytic materials. It highlights some of the warnings raised by experts in areas relevant to material engineering and photocatalysis, which are still widely disregarded in the literature. It aims at encouraging diligent practice in a field that has been described as a *landmine*, where every step must be taken carefully. The intention is not to dwell on a specific argumentative line since every aspect around the investigation of photocatalytic materials will deserve its own in-depth analysis. Instead, a case of study is presented as a form of *self-evaluation* to illustrate some of the main qualitative points highlighted here. The case of study is based on a photocatalytic disorder-engineered, hydrogen-doped TiO_2_, which has generated active research in many directions in the last decade.

## Qualitative Approaches for the Assessment of Photocatalytic Materials

### Synthesis Method

Guidelines of nanomaterial synthesis and the influence of synthesis parameters and conditions, as well as sample handling and storage, have been brilliantly discussed elsewhere (Baer et al., 2013). These authors raised awareness on the inadvertent steps that may lead to sample contamination and changes in material behavior. Their report included good practice in the preparation and organization of sample batches; control storage conditions such as light, humidity, temperature, and duration of storage; and attention to potential contamination from regular analytical tools, among other guidelines. Based on the lack of details in the synthesis description of most photocatalytic materials, this sort of code of good practice should be emphasized. Other qualitative measures with regards to the synthesis of novel photocatalysts concern useful information about failed synthesis experiments, assessment of method dependence, and scalability considerations. These details are often lacking in the literature but can greatly contribute to establish the reproducibility and best-performance conditions of novel materials.

#### Synthesis Mapping

**Figure 2:**
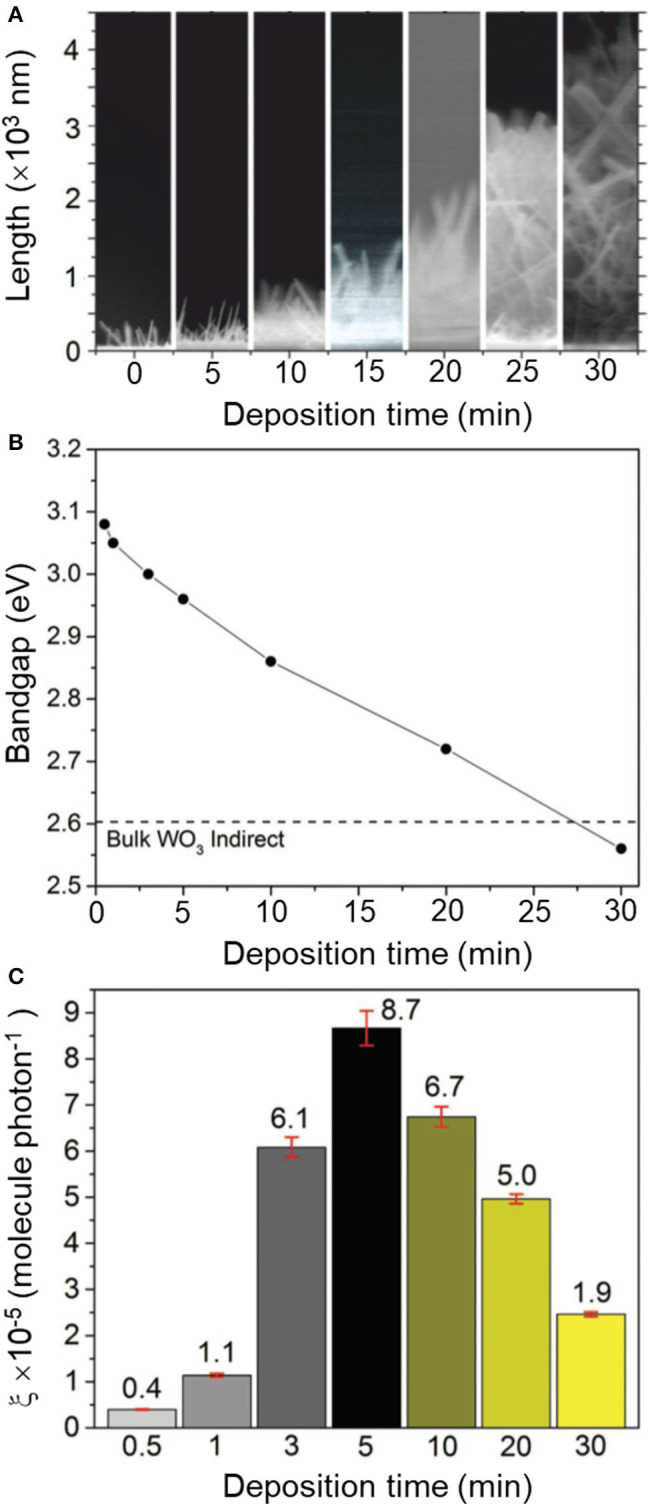
**(A)** Cross-section SEM images of non-stoichiometric WO_3−x_ thin films (400–4,500 nm) grown over 0.5–30 min (from left to right) via aerosol-assisted chemical vapor deposition (AACVD). Corresponding changes in **(B)** indirect bandgap (*E*_*bg*_) and **(C)** formal quantum efficiency (ξ, units molecules photon^−1^), given upon photodegradation of stearic (octadecanoic) acid molecules per incident photon. The dotted line in **(B)** corresponds to the bandgap of bulk WO_3_ (*E*_*bg*_ = 2.62 eV). Reproduced with permission from Ling et al. (2017).

Synthesis procedures are typically described for a single material with optimum functional properties, i.e., maximum photocatalytic activity. In order to establish correlation between material properties and photocatalytic performance, however, it is rather useful to follow the trace of experiments that led to the discovery of the best-performing sample. That involves reporting over a set of (widely regarded as) *negative* results with a broader description of experimental parameters affecting the material properties. It can be understood as *giving directions* to a particular destination as opposed to providing the address alone. It will guide groups in the synthesis of the optimized material, which may be particularly critical for strategies involving control over chemical composition and defect engineering, with crucial impact on photocatalytic properties (Tong et al., 2012; Bai et al., 2017, 2018). Instructive examples are often found in reports on solid solutions. For instance, changes in Ga/Al ratios in a series of β-AgAl_1−x_Ga_*x*_O_2_ materials resulted in the modulation of their corresponding bandgap energies, between 2.19 and 2.83 eV, and the identification of an optimized photocatalyst at *x* = 0.4 (Ouyang and Ye, 2011). In this case, analysis of material properties within the complete series between pure β-AgAlO_2_ and β-AgGaO_2_ allowed drawing conclusions about the origin of the photocatalytic activity of β-AgAl_0.6_Ga_0.4_O_2_ as due to an ideal balance between visible-light absorption and adequate redox potentials. Another interesting case concerned the growth of oxygen-deficient tungsten oxide (WO_3−x_) nanorods (Figure 2) using chemical vapor deposition (Ling et al., 2017). The authors observed a drastic change in bandgap energies ranging from 2.6 to 3.1 eV upon increasing reaction times within 30 s and 30 min. Yet, the trend of activities showed a non-linear behavior, with a maximum photocatalytic activity observed for the film deposited at *t* = 5 min (Figure 2). Analysis of this series of materials allowed establishing two regimes that contributed to the activity, with a balance between concentration of oxygen vacancies and changes in electronic structure.

#### Method Dependence

An unambiguous demonstration proving that a material is method independent is useful toward establishing a rationale for its photocatalytic behavior. Ideally, this demonstration will involve the successful synthesis of the material in different forms (powder, films) and following different synthesis approaches. It can also challenge the reproducibility of the chosen approach and provide information about its potential scalability. Following a single procedure, the potential influence of reactor elements must be carefully considered. A study on the synthesis of disorder-engineered, hydrogenated TiO_2_–used as a case of study below—showed different optical properties, either black or blue products, from otherwise similar synthesis conditions using either stainless steel or quartz reactors, respectively (Danon et al., 2012). The sensitivity of this material to minor changes in precursor gas flows, for example, could drastically influence the hydrogen content or presence of oxygen vacancies in the final product. In addition, the authors noticed the presence of small amounts of chromium as an impurity in the sample obtained from the stainless steel reactor, which they attributed as responsible of its darker color. Thus, replacing system elements can contribute toward an understanding of material physical and functional properties.

It is also important to account for the potential influence of the substrate on the physical and functional properties of supported materials. This is obviously well-understood in specific synthesis designs where the influence of the substrate is expected, for example during epitaxial growth of films on single-crystal surfaces using atomic layer deposition. It is less obvious whenever the influence of the substrate is accidental, for example during the template-induced growth of a particular crystalline phase under fast-growing regimes by chemical vapor deposition. This effect has been observed, for instance, in the unexpected deposition of rutile TiO_2_ films under relatively low-temperature conditions over a rutile substrate (Quesada-Cabrera et al., 2014b). Substrate-induced changes in optical properties have also been observed in vanadium oxide (VO_2_) films deposited on tin-oxide-based supports (Powell et al., 2017). In this case, the presence of electron-withdrawing substituents such as fluorine contributed to the oxidation of V^4+^ ions in the crystal lattice, with impact on the thermochromic properties of the films.

#### Scalability Considerations

Studies exploring material physical and functional properties upon scaling up synthesis conditions are still rare in photocatalysis. In terms of material synthesis, scalability considerations are often referred to the use of a particular industrial synthesis method or the configuration of systems based on abundant, inexpensive materials. It would be desirable to establish comparison of physical and functional properties of advanced photocatalysts upon laboratory- and large-scale production. For example, a case of study compared the properties of a Zn–Ce oxide nanoparticle system obtained upon different volume regimes (laboratory and pilot plant, respectively) under continuous supercritical-water conditions (Tighe et al., 2013). In that case, the physical properties of these nanomaterials were closely comparable, with similar solubility of Zn (20 mol%) in the CeO_2_ (fluorite) lattice. But perhaps more relevant to experts in material engineering is to consider the expected translation of functional properties upon scaling up from the *nano-* to the *macro*scale. Discussion on some key size-dependent phenomena in photocatalysis, such as charge transport, has been presented previously (Tomkiewicz, 2000). Essentially, it takes into account issues related to interparticle charge hopping and the different pathways of transport between nano- and microcrystalline materials. The author also highlighted that the two parameters of Langmuir isotherm—widely used to describe adsorption properties—namely, adsorption coefficient and the maximum number of molecules per unit surface area are likely size and structure dependent in mesoscopic materials.

### Material Properties and Characterization

The challenges involved in any attempt to correlate material properties and activity have been raised by world-leading experts in the field (Ohtani, 2017). Taking the doping of materials as an example, it is tempting to attribute an enhanced efficiency to electronic properties induced by the introduction of the dopant; however, this claim cannot be raised unambiguously if the doping process affects other key parameters in the material, such as its surface microstructure. Essentially, any modification to a pure material will likely affect several intrinsic properties, and isolating the impact of a single parameter to the observed photocatalytic activity is arduous, to say the least. Whenever possible, it is important to determine the limits of the property claimed as responsible for the photocatalytic enhancement, which is likely to follow a non-linear trend. For instance, in a heterostructure with a buried heterojunction between two semiconductor layers, the balance between light absorption and charge carrier diffusion length upon layer thickness will delimit a maximum point in photocatalytic activity beyond which any potential synergistic advantage from forming the heterojunction will be canceled by charge recombination. Knowing the range of activities within these constrictions will contribute to draw conclusions about the origin of the photocatalytic behavior of the material.

Issues related to an inadequate or incomplete characterization of nanomaterials have been widely discussed (Baer et al., 2013). These issues mainly arise from the multidisciplinary nature of the area of materials chemistry and the lack of access to specific methods for most groups. In most cases, it is only possible to establish the significance and promising opportunities of a novel material once the main tasks of its characterization have been fulfilled satisfactorily across several groups worldwide. Here, sample reproducibility plays a crucial role. In fact, minor differences in lab-synthesized samples are often at the center of controversy around the literature on functional materials. It is important to understand the dynamic nature of nanomaterials and how they are influenced not only by synthesis parameters but also by environmental conditions (Baer et al., 2013). These may change physical properties such as particle size, shape, and structure, even across the same sample batch, or chemical properties, with changes in atomic oxidation states, surface segregation, or the stability of different surface groups. All these changes may have significant impact on key phenomena in photocatalytic behavior, such as light absorption and charge transport properties, adsorption/desorption kinetics, or surface deactivation. In addition, the functional properties of nanoparticles are also affected by *proximity* effects, arising from particle–particle (or particle–substrate) interactions, which may induce charging or coupling of quantum states, for example in novel plasmonic photocatalysts.

#### in situ/Operando Techniques: Structure–Function Correlation

The art of establishing correlation between material properties and photocatalytic activity could be framed as *abstract expressionism*, considering the number of interrelated elements contributing to the overall picture. Focusing on a single element as responsible for a sudden increase in photocatalytic activity—for instance, the electronic synergy upon formation of a heterojunction—may come at the expense of ignoring other influencing properties, such as an associated change in surface area or surface composition. The application of qualitative measures is particularly important here in order to minimize the range of interpretations for a given set of observations. Not surprisingly, leading experts (Ohtani, 2017) have emphasized the pitfalls of establishing causal relations for photocatalytic activity. This problem is rooted in the common practice of performing characterization analysis *ex situ*, which involves assumptions related to the homogeneity of the sample (similar ratios of crystalline and amorphous phases; similar nature and concentration of defects across the sample); chemical stability (no changes in oxidation states of surface species or in the concentration of oxygen vacancies during reaction conditions); and surface inertness (similar surface acidity; absence of sensitization through electron transfer at the catalyst–substrate interface). Instead, a more accurate interpretation of the *action painting* of a semiconductor can be established using *in situ* techniques with in-depth material characterization during irradiation, i.e., while the photocatalyst is in an excited state, and ideally under reaction (*in operando*) conditions. This approach is widely established in thermal catalysis (Newton and van Beek, 2010; O'Brien et al., 2011; Beale et al., 2013, 2014; Liu et al., 2015) and is rapidly emerging in photocatalysis (Bora et al., 2013, 2014; Dong and Vayssieres, 2018; Tan et al., 2019). A review on dedicated synchrotron X-ray microprobes and nanoprobes for *in situ*/*operando* material characterization has been reported recently (Mino et al., 2018).

*In operando* photocatalytic studies have been traditionally used to investigate reaction mechanisms using infrared spectroscopy (Rohmann et al., 2008; Renckens et al., 2010; Hauchecorne and Lenaerts, 2013; Zandi and Hamann, 2016). In the last decade, *in operando* analysis of the photocatalyst surface has been widely carried out using advanced (time-resolved) X-ray spectroscopy techniques, with emphasis into photoelectrochemical water splitting and hydrogen evolution processes (Braun et al., 2012; Bora et al., 2013; Crumlin et al., 2015; Baran et al., 2016; Li et al., 2016b; Neppl et al., 2016; Lu et al., 2020). These techniques are not without their own experimental and analytical limitations. The use of a high-energy X-ray beam may induce sample damage, and there are many experimental factors that can affect the resolution of the data collected, including sample preparation and the type of cell design chosen for a particular reaction (Jacques et al., 2009). These authors noticed the sensitivity of the data to the experimental setup, specifically the impact of catalyst sieve fraction size and beam position on the quality of the data. Fitting issues and misinterpretation of the data are a common source of controversy in X-ray spectroscopy studies. Experts in the area have raised concerns, for example, about cases where a change in edge position has been wrongly assigned as due to a change in oxidation state without consideration of the ionicity of the local environment that may also contribute to this effect (Beale et al., 2009; Agote-Arán et al., 2019a,b). These issues may also be aggravated upon the complexities of time-consuming analysis considering the amount of data that can be obtained upon second/subsecond time resolution (Jacques et al., 2009).

Access to synchrotron techniques is still restricted, and the use of specialized equipment and purpose-built instrumentation is not universally accessible. Progress has been made toward developing lab-based *in situ*/*operando* approaches for the analysis of photocatalytic materials using, for instance, advanced laser techniques (Pastor et al., 2017) or *in situ* nuclear magnetic resonance (Wang et al., 2016). Even as core techniques applied *ex situ*, qualitative measures must be considered, since an inadequate analysis based on these techniques is still a source of frustration and controversy in the literature.

#### Core Characterization Techniques

Most analytical techniques would require individual dedicated reviews to compile an exhaustive list of qualitative measures for an optimum analysis of samples. This is particularly the case for specialized methods relevant to photocatalytic materials, such as transient absorption spectroscopy or electrochemical techniques. These methods are usually operated by experts in specialized groups, and their discussion is outside the scope of this review. There is, however, a set of *core* analytical tools (Baer et al., 2013), available to most material synthesis laboratories, that may be used for recurring characterization and can even be adapted for *in situ*/*operando* analysis, as long as qualitative measures are ensured for their adequate operation and an understanding of their limitations.

##### Structural analysis

X-ray diffraction (XRD) is the most common core technique for phase identification and structural information of materials. Several issues have been raised with regards to the limitations of XRD analysis and the interpretation of XRD patterns (Ohtani, 2010; Hargreaves, 2016). The consideration of a material as *crystalline* from inspection of diffraction patterns—based on the sharpness of diffraction peaks—is venturous, and account of amorphous parts and density of crystalline defects is largely excluded. Yet, the presence of defects or amorphous parts can play an important role in the performance of photocatalytic materials (Yu et al., 2013; Quesada-Cabrera et al., 2014b; Sivula, 2014; Tian et al., 2015; Zhao et al., 2015). The presence of traces of impurity phases on crystalline substrates may be unnoticed, particularly if the sample contains amorphous parts that can raise the baseline of the XRD pattern. These impurities can have significant impact in photocatalytic performance. Common lab-based XRD instruments will not account for potential structural differences between surface and bulk domains. This is particularly relevant to photocatalytic materials, where any attempt to establish structure–function correlation will rely on an accurate description of surface physical properties.

Beyond technical limitations, it is still common practice to use diffraction peak broadening to estimate *crystallite size* using the Scherrer equation with little attention to its associated restrictions. The Scherrer equation (*d*_*hkl*_ = *K*λ/β_*hkl*_cosθ) allows for an estimation of the coherent diffraction domain size in the direction perpendicular to the lattice planes with Miller indices *hkl*, using a numerical (crystallite shape) factor K and the full-width at half-maximum (β_*hkl*_) and Bragg angle (θ) of the corresponding diffraction peak for a given wavelength (λ). Since the analysis is sensitive to a particular direction, special care must be paid in the case of highly anisotropic crystallite morphologies, such as needles or disks (Qazi et al., 2009). The Scherrer equation was derived for the ideal condition of a parallel, infinitely narrow and monochromatic beam incident on a monodisperse powder of cube-shaped crystallites (Holzwarth and Gibson, 2011). It is important to notice that the K factor is dependent on features such as crystal morphology, the specific reflection used in the analysis, its integral breadth or full width at half maximum, and sample inhomogeneity through coherently diffracting size distributions (Hargreaves, 2016). A key challenge in this analysis is elucidating the component of peak width as due to the material itself, with additional components arising from strain or disorder, instrumental broadening, and the use of non-monochromatic X-ray sources. Although most advanced photocatalytic systems are based on nanomaterials, it is also worth noting that the Scherrer equation is also restricted to average sizes of up to 100–200 nm, since peak broadening decreases with increasing crystallite size and the sample-broadening component cannot be isolated from other broadening effects (Hargreaves, 2016).

Vibrational spectroscopy, particularly Raman spectroscopy, is widely used as complementary technique to XRD methods for structural analysis. Raman spectroscopy can be conveniently used as a fast, *in situ* surface technique (Newton and van Beek, 2010), and it can be highly sensitive to minor structural changes, such as the incorporation of dopants in the semiconductor lattice (Mazzolini et al., 2016). Some of the experimental limitations of this technique are related to potential fluorescence or sample degradation upon laser radiation. In terms of qualitative measures, however, particular attention should be paid to the analysis and interpretation of Raman spectra, as gathered from many reports in the literature. It is not uncommon to find baseline-subtracted spectra across the literature without access to the original data. The Raman baseline may contain useful information about amorphous parts in the sample, and the subtraction process can lead to artifacts in the spectrum. The assignment of Raman bands is not straightforward, particularly whenever there is significant band broadening, and care must be taken to avoid an overinterpretation of the data. Meticulous Raman studies can provide information on sample morphology, surface roughness, and crystallite size (Ekoi et al., 2019).

Further core characterization techniques for structural analysis include scanning/transmission electron microscopy (SEM/TEM) and atomic force microscopy (AFM). The main advantages and limitations of these techniques have been extensively reviewed (Linkov et al., 2013). Electron beam damage may introduce potential artifacts in SEM/TEM analysis. SEM requires high vacuum conditions and good electroconductivity of surfaces for high-resolution analysis. Semiconductor materials, such as TiO_2_, are often coated with ultrathin conducting films—typically via sputtering of carbon or gold nanoparticles; however, this treatment may damage or deform the sample. Sample preparation can be laborious for TEM analysis, which requires thin enough samples for good contrast. Differences in contrast arise also from the nature of materials—since heavier atoms scatter electrons better than lighter atoms—and the orientation of crystalline domains. In the case of AFM, imaging artifacts may arise depending on the tapping mode of the cantilever tip. Examples of studies have been shown where estimated nanoparticle size was related to the oscillation amplitude and curvature of the tip, as well as the physical properties of the sample (Linkov et al., 2013). AFM has been used to estimate surface roughness of films as a means of evaluating differences in surface areas among photocatalytic materials. In general, the region of analysis in these techniques is very small, and the mapping of several spots across the entire sample is required in order to establish significant differences in morphology and particle aspect.

Surface area is a dominant feature within the collective contributions of physical properties in both catalytic and photocatalytic materials. As a simple approach, surface area will promote the adsorption of substrates and the promotion of key electron transfer processes. In addition, surface roughness may also contribute to efficient light absorption (Osterloh, 2013) and thus further promote quantum efficiency in this sense. Analysis of surface area is widely provided when using powders, since the Brunauer–Emmett–Teller (BET) approach, based on the adsorption of inert gases, is well established for these samples. In contrast, surface area analysis of thin films can be more challenging as it requires more specialized equipment. Related methods involve krypton adsorption at liquid–nitrogen temperatures (Krause et al., 2010) or the use of surface acoustic waves (Ricco et al., 1989). Attention to changes in specific surface area and morphology is crucial when comparing samples with reference materials.

##### Surface composition

X-ray photoelectron spectroscopy (XPS) is a powerful and versatile lab-based core tool for the analysis of surface composition. This technique provides qualitative and quantitative chemical information from the near surface region of films and particles, and thus, it is of fundamental importance in the characterization of catalytic materials (Venezia, 2003). The limitations of XPS include the need of adequate sample preparation and sample handling, as well as operating environments under high vacuum conditions (Baer et al., 2013). The analyzed region in lab-based instruments is relatively large (typically ~400 μm), and analysis time scales are rather long. Most XPS instruments allow for analysis along depth distribution profiles upon argon etching of the surface, but this is obviously a destructive approach and it may lead to changes in oxidation states of the species under analysis. In the analysis of catalytic systems, XPS can provide useful information about the electronic and morphological structure of materials; dispersion of supported catalysts and surface segregation of relevant species (Venezia, 2003); and even phase quantification using valence band analysis (Breeson et al., 2017). The use of appropriate modeling and relative sensitivity factors (RSF) must be stressed in the quantification of XPS spectra, as it seems to be the most common source of misinterpretation of the data. A challenge in analysis of XPS data is that often spectra have complex shapes, caused by overlapping features. It then becomes necessary to fit a model to the data to extract the necessary information. A common area of difficulty is in fitting a physically realistic model to the experimental spectra. It is important to consider how to constrain the model according to the known physical relationships between spectral features; otherwise, it is easy to come to incorrect conclusions.

##### Optical analysis

**Figure 3:**
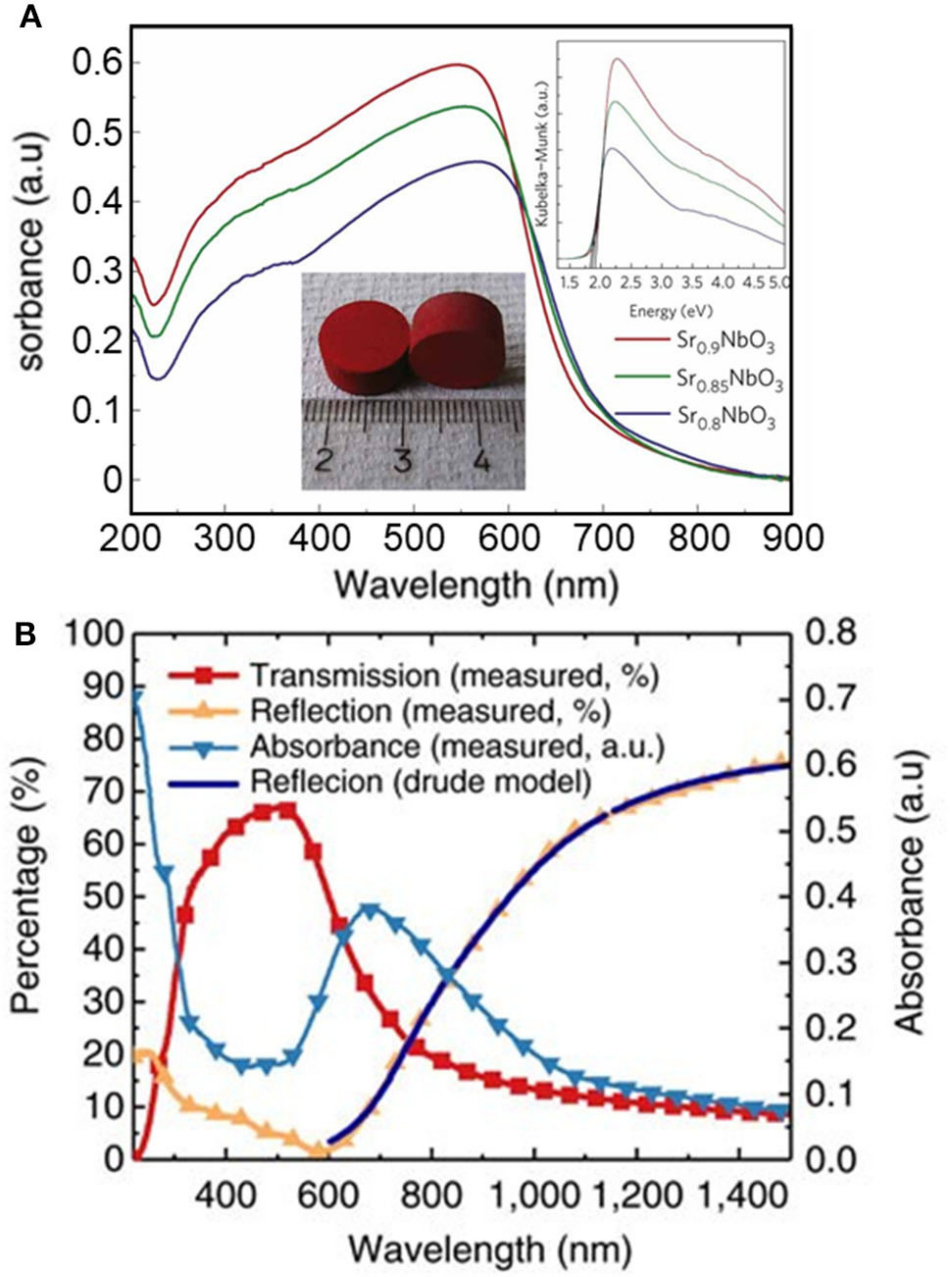
**(A)** Ultraviolet–visible absorbance spectra (converted from diffuse reflectance spectra) of Sr_1−x_NbO_3_ (*x* = 0.1, 0.15, and 0.2). Top inset shows the corresponding Kubelka–Munk transformation of the absorption curves. Typical sintered pellets (Sr_0.9_NbO_3_) are shown in the bottom inset. Reproduced with permission from Xu et al. (2012). **(B)** Optical properties of a Sr_1−x_NbO_3_ film deposited at oxygen partial pressure of 5,106 Torr from spectroscopic ellipsometry and the Kramers–Kronig transform. The accurate optical analysis corrected previous electronic interpretations and identified the compound as a plasmonic photocatalyst. Reproduced from Wan et al. (2017) (open access).

The optical properties of photocatalytic materials is widely studied using spectrophotometry. The optical bandgap is typically estimated using the Tauc method from absorbance analysis (Murphy, 2007; Viezbicke et al., 2015). The convolution of absorption spectra can be challenging, particularly when using transparent thin films deposited on substrates of variable composition, such as glass slides. It is worth noting the intrinsic limitations of the Tauc method, originally intended for use with amorphous materials, where the presence of localized energy states is largely assumed. The method is inappropriate for analysis of degenerately doped bulk materials, for example, since high doping levels can give rise to a band tail rather than to localized energy states (Dolgonos et al., 2016). It is also important to note that absorption-based spectroscopic methods are basically a bulk technique, considering the large penetration depths of photons in the absorption range of most semiconductors (Dolgonos et al., 2016). Therefore, any surface electronic processes giving rise to color centers or the formation of surface plasmon resonance may be hidden from optical analysis using spectrophotometry. Very recently, the identification of a red metallic semiconductor material (Figure 3A) as a plasmonic photocatalyst (Wan et al., 2017) changed the original interpretation of its unique electronic properties (Xu et al., 2012). The misconception emerged from a misuse of the Tauc method and the Kubelka–Munk transformation, which overlooked the plasmonic effect. Instead, the authors advocated for the use of spectroscopic ellipsometry and the Kramers–Kronig transform of reflectance data (Figure 3B).

##### Theoretical approaches

An adequate synergy between theoretical and experimental studies is crucial toward the development of a new generation of novel, efficient materials for widespread use (Stevanović et al., 2014; Sachs et al., 2018; Wang et al., 2019; Davies et al., 2020; Doherty et al., 2020). A set of qualitative measures should include an evaluation of how experimental analysis influence on the effective development of an underlying theory and to define the limitations and constrictions bound to first-principle calculations. This is not always clear when constructing an interpretation for a set of experimental observations. Density functional theory (DFT) is a widely used, powerful tool to investigate the electronic structure of semiconductors, but the extent of inaccuracies in the application of DFT is unstated in most theoretical studies (Pacchioni, 2015). It will provide, for instance, crucial information regarding the position of band edges of a single material in the vacuum. Hence, the relative band structures of two individual semiconductors forming a heterostructure—but *not* in contact—can be compared. A more relevant picture from an experimental viewpoint would include the equilibration of the fermi level over the interface and, particularly, the fate of this fermi level in equilibrium into quasi-fermi levels under illumination. This information is, however, out of reach for DFT calculations. Further insight in this sense may be sought from complementary computer-aided design methods together with advanced characterization techniques, such as transient absorption spectroscopy (Iqbal et al., 2016; Iqbal and Bevan, 2018a,b; Sun et al., 2019).

### Photocatalytic Performance

The main qualitative measures around the functional assessment of photocatalysts concern the choice of adequate tests under appropriate irradiation conditions, knowledge of photon emission rates and spectra, and the application of relevant control experiments, among other factors.

#### Control Experiments and Blank Samples

Control experiments are key toward establishing the nature and appropriate quantification of a photocatalytic process. Yet, it is still surprising to find broad claims of enhanced photocatalytic activity without attention to adequate control tests. These include processes in the absence of substrate or photocatalyst (*procedural* blanks) or any sacrificial agents (*reagent* blanks) used in the experiments. It is also important to gather information of the process in the dark in order to rule out potential reactions driven by chemically active surfaces. Whenever possible, it will also be useful to study the behavior of visible light-active photocatalysts irradiated under wavelengths outside the material absorption range, as discussed for action spectra analysis below.

The use and purpose of *blank* and *reference* samples are often confused in the literature on photocatalytic materials. Commercial photocatalytic products such as *Evonik P25* or *Pilkington's Activ* glass have been widely used as reference or standard samples (Mills et al., 2003; Ohtani et al., 2010). These materials contribute to establish the suitability of the experimental procedures and to frame the efficiency of the material within a broader context, effectively allowing comparison with other novel photocatalytic systems. Claims of enhanced activity can be hardly raised, however, upon comparison with materials of very different properties to those used as reference samples. In this case, *blank* materials, typically parent materials obtained via similar synthesis steps to those under study, should be used in addition to reference materials. It is also interesting to carry out experiments using blank samples with similar physical properties but otherwise inactive under given irradiation conditions.

#### Dye-Sensitized Processes

**Figure 4:**
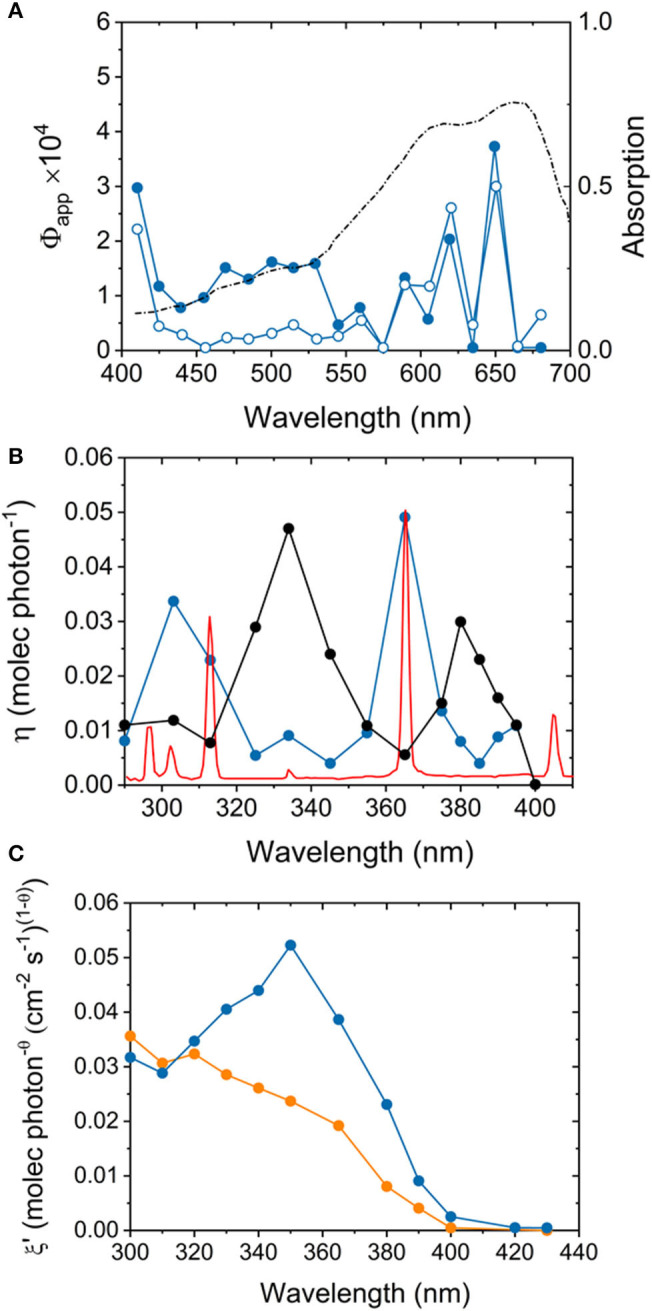
**(A)** Action spectra of sulfur-doped TiO_2_ (full symbols) and commercial TiO_2_ *Evonik* P25 (empty symbols) during methylene blue decomposition under visible light irradiation. The diffuse reflectance spectrum of adsorbed methylene blue is included for reference. The plot highlights the activation of the dye-sensitized process under these irradiation conditions. Adapted with permission from Yan et al. (2006). **(B)** Action spectrum of *Evonik* P25 TiO_2_ (black line) during photodegradation of 4-chlorophenol as reported by (Emeline et al., 2000), generated using the emission spectrum of a 1,000 W Xe/Hg lamp (blue line). A normalized, high definition emission spectrum of a 1,000 W Xe/Hg lamp is illustrated for reference (red line). Ignoring the sharp emission lines of the light source, the estimation of quantum efficiencies gives rise to an erratic curve consisting of peaks and troughs, which lead to misleading conclusions over the spectral sensitivity of the sample. Adapted with permission from Lee et al. (2017a). (**C**) Modified action spectra plots of commercial carbon-modified TiO_2_ (Kronos VLP 7,000, blue line) and *Evonik* P25 (orange line) during photodegradation of stearic acid, highlighting the impact of a photosensitized process driven by surface carbonaceous species. Adapted with permission from Quesada-Cabrera et al. (2014a).

A recent review (Minella et al., 2017) acknowledged that over 75% of the claims reporting the photocatalytic degradation of organic pollutants using hybrid materials were based on the discoloration of dyes as standard substrates. The authors rightly excluded these studies from their examination based on potential *problems of interpretation*. This is despite the many voices warning about dye-sensitization mechanisms upon electron injection into solids, which hinder any conclusions about the *intrinsic* photocatalytic properties of the semiconductor (Yan et al., 2006; Ohtani, 2017; Lee et al., 2018; Sáenz-Trevizo et al., 2019). It is still surprising to find claims of visible light activity using methylene blue (MB) under irradiation conditions within the absorption range of the dye (Figure 4A). The correct procedure and limitations of this test have been meticulously detailed by experts in the field (Yan et al., 2006; Lee et al., 2018). An ISO test based on the photoreduction of resazurin dye is also available now for the assessment of photocatalytic materials (International Organization for Standardization., 2018). Many other ink-based tests have been designed as screening methods and for the rapid and remote assessment of photocatalytic self-cleaning materials (Mills et al., 2013, 2014a,b, 2016, 2017; Mills and Wells, 2015). Any claims of visible light activity using visible-light absorbing dyes within the emission range of the light source cannot be raised unambiguously.

#### Probing Visible Light Activity: Action Spectrum

Most claims of visible light activity are based on observations of photocatalytic behavior using white light sources or solar simulators such as Xe arc lamps with cutoff filters at λ > 400–420 nm. Detail information about the type of filter used is often missing, but their efficiency to block high-energy photons can differ significantly (Dunnill, 2014). This transmission tail may activate efficient UV-absorbing photocatalysts and thus lead to ambiguous conclusions about their photocatalytic ability in the visible range. The current authors raised this issue during photocatalytic studies involving nitrogen-doped TiO_2_ films (Quesada-Cabrera et al., 2017). Control experiments using wide-bandgap, reference materials such as TiO_2_ are crucial in this sense, since they are inactive under visible light. The photocatalytic activities of the materials in the UV range should be reported as part of the control experiments, since any similar trends under the two irradiation regimes may reveal the leaking of high-energy photons. It is also worth noting that the efficiency of cutoff filters tends to drop with usage, yet the emission spectrum of the light source with the filter in place is seldom reported. The emission spectrum should be obtained from an efficient, calibrated spectroradiometer, whenever possible.

A more appropriate analysis of the intrinsic photocatalytic properties of a semiconductor material can be carried out following its action spectrum, which accounts for photonic efficiency, η, as a function of excitation wavelength, λ (Lee et al., 2017b). The action spectrum is expected to follow the light absorption profile of the photocatalyst, and thus, it can help identify any potential artifacts or photosensitizing processes in the system. Early studies observed an erratic trend of peaks and troughs in the action spectrum of TiO_2_ during photodegradation of 4-chlorophenol (Emeline et al., 2000), which led to drawing conclusions about the spectral sensitivity of the catalyst. Recently, however, careful action spectrum studies (Lee et al., 2017a) revealed that the work had neglected the emission lines of mercury from the Xe/Hg lamp used in the original experiments (Figure 4B). Similarly, the action spectrum of a carbon-modified TiO_2_ commercial product (*Kronos VLP 700*) (Orth-Gerber and Kisch, 2005) showed a trend (Figure 4C) inconsistent with the absorption spectrum of the pristine material (Quesada-Cabrera et al., 2014a). The study demonstrated that the allegedly visible-light activity of the commercial photocatalyst was due to a photosensitized process, presumably from surface carbonaceous impurities (Zabek et al., 2009). It was noticed that these species would degrade under prolonged solar irradiation, thus rendering the material inactive in the visible range. Similar photosensitization processes from surface species have been identified for TiO_2_-based hybrid photocatalysts combined with graphitic carbon nitrides (Ladva et al., 2017) and nitrogen-doped systems (Quesada-Cabrera et al., 2017).

### Material Stability

The evaluation of photocatalytic materials requires of an assessment of action and stability under broad reaction conditions; this is, however, often missing in the literature. For example, information regarding the photostability of visible light-active materials under prolonged UV irradiation is necessary in order to establish their potential for outdoor applications. Cycling experiments are typically carried out as a means to evaluate the stability of a material. While this approach is useful, it is important to note that the recovery of the sample or its performance over a limited number of cycles does not prove that the process is photocatalytic (Childs and Ollis, 1980). It is also worth noting that the action assessment during cycling experiments for a given process is not transferable to any other process under different conditions. Information about the sample recovery procedure after each cycle test should be clearly stated. The recovery procedure may involve removing residual solutes to minimize deposition of salts or other species; removing solvent in way that minimizes particle aggregation and any potential alteration of particle phases, as well as interference with analytical instruments; avoiding or minimizing the dissolution or abrasion of surface coatings; and avoiding chemical reactions with the medium or its contaminants (Nurmi et al., 2011). Different particle extraction processes have been discussed elsewhere (Baer et al., 2013). Any changes in activity during cycling tests, either negative or *positive*, i.e., involving a decrease or *increase* in activity (Sorathiya et al., 2016), will be indicative of photocatalyst instability. After the cycling tests, full characterization of the material must be carried out without further posttreatment or cleaning of the sample surface that may alter its original physical properties. Since the time scales of material aging can range from seconds to years, it is useful to carry out cycling experiments throughout considerable length of times, stating these periods clearly. For example, previous work demonstrated the efficiency of core–shell WO_3_/TiO_2_ films after a period of 1 year since their original synthesis (Sotelo-Vazquez et al., 2017).

## Case of Study: Disorder-Engineered, Black TiO_2_

**Figure 5:**
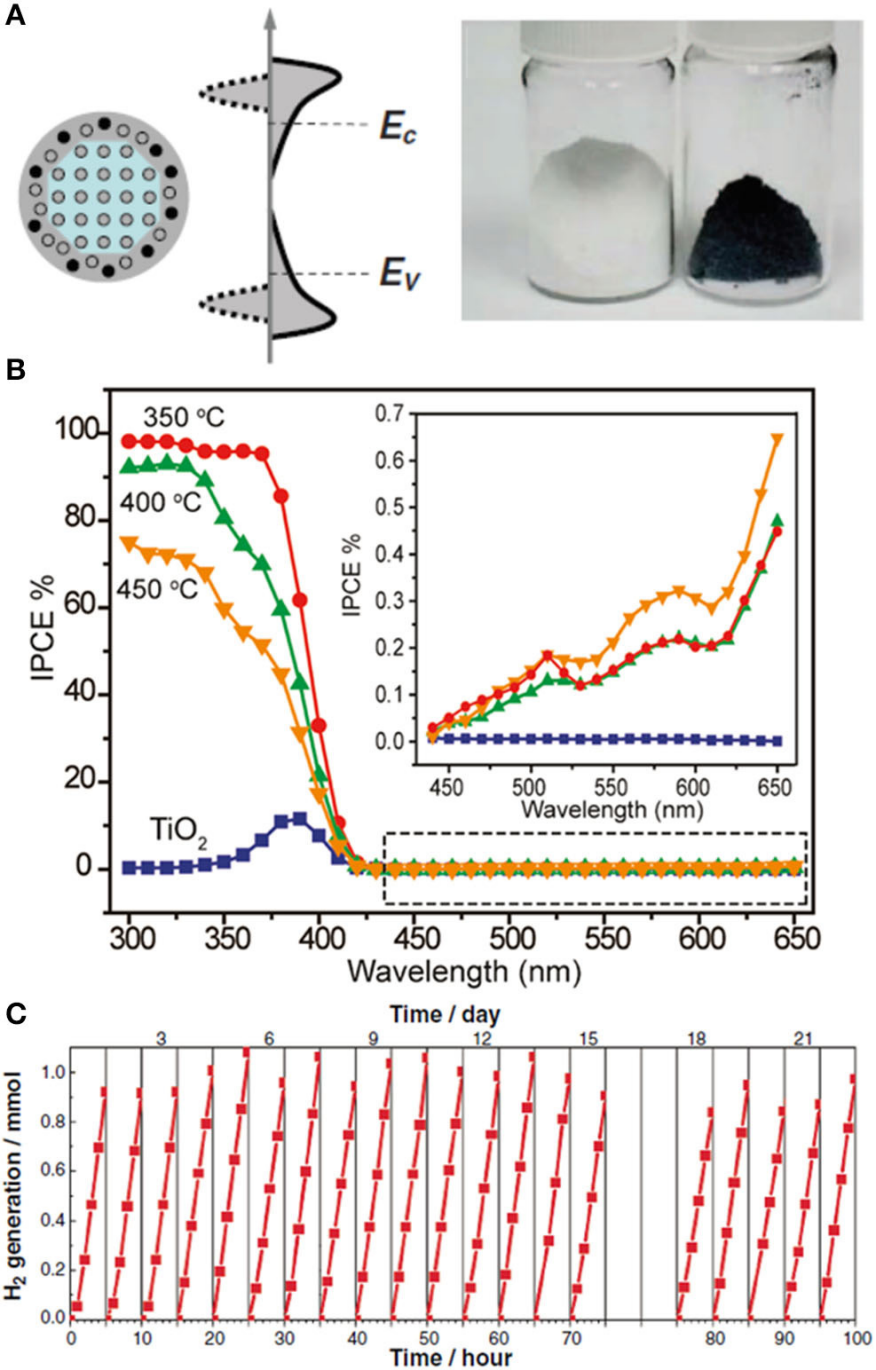
**(A)** Schematic illustration of the structure and electronic DOS of a disorder-engineered semiconductor nanocrystal with dopant incorporation. Dopants are depicted as black dots, and disorder is represented in the outer layer of the nanocrystal. The conduction and valence levels of a bulk semiconductor (E_C_ and E_V_, respectively) are shown, together with the bands of the nanocrystals (left-hand side). The effect of disorder, with broadened tails of states extending into the otherwise forbidden band gap, is also shown (right-hand side). Photograph of unmodified white and disorder-engineered black TiO_2_ nanocrystals. **(B)** Incident-photon-to-current-conversion efficiency (IPCE) of disorder-engineered hydrogen-doped TiO_2_ nanowires prepared at different temperatures (350, 400, and 450°C), collected at a potential of 0.6 V vs. Ag/AgCl. Inset: highlighted region in the dashed box at incident wavelengths between 440 and 650 nm. **(C)** Cycling experiments showing hydrogen production from photocatalytic water splitting using black TiO_2_ nanocrystals under simulated solar light. Reproduced with permission from Chen et al. (2011). Reproduced with permission from Wang et al. (2011).

The engineering of TiO_2_-based materials has always received great attention in photocatalysis, as they offer a real chance to reach the global market. The discovery of a hydrogenated, black TiO_2_ material, reported early last decade (Chen et al., 2011), with over 4,000 citations so far, encouraged research across many disciplines, including catalysis, batteries, supercapacitors, field emission, fuel cells, and microwave absorption (Chen et al., 2015; Wang et al., 2017; Yan et al., 2017; Ullattil et al., 2018). The short history of black TiO_2_ is, however, troublesome, with conflicting results around the nature of its structural features and the origin of its unique properties (Rajaraman et al., 2020). The original work described the synthesis of a disorder-engineered, hydrogenated TiO_2_ material (Figure 5A), designed as a core–shell nanoparticle with a crystalline anatase core and a highly disordered, hydrogen-doped shell (Chen et al., 2011). It was produced after hydrogenation of anatase particles under 20-bar H_2_ atmosphere at 200°C for 5 days. The authors did not provide insight into the specific conditions of this synthesis (*Synthesis Mapping*) or a broader range of conditions and how these would affect the material properties (*Method Dependence*). Unfortunately, minor modifications in the synthesis procedure seem to have a critical impact in its physical and functional properties, as discussed below. The potential scalability of this material has not been addressed (*Scalability Considerations*).

A wide range of appropriate characterization techniques (*Material Properties and Characterization*) has been used to study black TiO_2_ including *in situ* advanced techniques (*In Situ/Operando Techniques: Structure–Function Correlation*) such as X-ray absorption/emission spectroscopy (XAS and XES, respectively) as well as core techniques such as X-ray diffraction, Raman spectroscopy, and nuclear magnetic resonance (Chen et al., 2013) (*Core Characterization Techniques*). For almost all hydrogenation approaches, disordered structural features were observed near the surface of black TiO_2_ with a general lattice contraction of the disordered layer, although expansion of the shell has also been reported (Chen et al., 2015). Within these samples, the presence of Ti^3+^ species, oxygen vacancies, and Ti–OH and Ti–H groups seems arbitrary (Chen et al., 2015). In the original work, the observation of broad features in the Raman spectrum of their black TiO_2_ sample led to an ambiguous claim about the breaking down of selection rules for this sample (*Structural Analysis*). Most of these features have not been observed in subsequent work, and emerging bands and band shifting in some cases have been attributed to other factors, such as phonon confinement, the presence of oxygen vacancies and other TiO_2_ phases, among others (Ullattil et al., 2018). The role of the disordered layer is also controversial, and it is not clear how it participates in the photocatalytic properties of the material (Diebold, 2011; Zhang and Park, 2016). Comparative structural analysis (Xia and Chen, 2013) have provided quantitative insight into the size, shape, and crystalline facets of the TiO_2_ nanocrystals. The study concluded that hydrogenation causes a reduction in the crystalline phase compared to the parent material, and it exposes different facets in the black modification. The authors speculated whether these structural changes may contribute to the high activity of black TiO_2_. In light of all the controversy around these materials, many authors have advocated for further in-depth characterization at atomic scale using a range of advanced techniques such as scanning tunneling microscopy (STM), scanning transmission electron microscopy (STEM), X-ray absorption fine structure (XAFS), and X-ray absorption near edge structure (XANES) (Zhang and Park, 2016).

The interest on disorder-engineered, hydrogenated TiO_2_ materials has been particularly stimulated due to their optical properties (*Optical Analysis*), although not all hydrogenation procedures result in black TiO_2_ (Liu et al., 2016; Ullattil et al., 2018). There has been active discussion around the origin of the broad visible light absorption of this material from UV to the near-infrared region (Diebold, 2011; Chen et al., 2013, 2015; Liu et al., 2013; Zhang and Park, 2016). The intense yellow and blue colors observed in other reduced modifications were attributed to the incorporation of valence band-edge states due to Ti^3+^ species; however, these species were not detected in the original black TiO_2_ version (Chen et al., 2013). Instead, the authors attributed its optical properties to disorder-induced band tail states, forming a continuum extending to, and even overlapping with, the conduction band edge. These energy states, combined with dopant energy levels, would become centers for optical excitation and relaxation processes. The authors also noted the potential role of charge trapping sites in the disordered layer, preventing fast recombination and promoting electron transfer and photocatalytic efficiency. DFT calculations (*Theoretical Approaches*) indicated that the lattice disorder in black TiO_2_ would come from the interplay between surface Ti–O bonding breaking upon adsorption of hydrogen, with formation of Ti–H and O–H bonds, and the desorption of hydrogen molecules in the bulk (Liu et al., 2013). As a result of this disorder, strong localized mid-gap states would shift the valence band maximum to higher energies while the conduction band minimum remained unchanged. This shift in the valence band is, however, not always observed in black TiO_2_ (Chen et al., 2015).

The photocatalytic performance of black TiO_2_ is also not absent of controversy (*Photocatalytic Performance*). At this point, it is worth noting that broad light absorption does not necessarily correlate with extended photocatalytic activity. The initial study (Chen et al., 2011) referred to the change in optical properties of black TiO_2_ as a *dramatic color change* with substantial enhancement of *solar-driven* photocatalytic activity. It is tempting to correlate this enhanced activity and the extended light absorption of black TiO_2_ in the visible range; however, as pointed out by some of these authors (Wang et al., 2017), the photocatalytic performance of the material in the visible range was *unsatisfactory*. Instead, the enhanced activity of the original modification is restricted to the UV range, and it is not always observed in other hydrogenated forms of TiO_2_ (Rajaraman et al., 2020). The original work (Chen et al., 2011) demonstrated the photocatalytic properties of black TiO_2_ upon hydrogen production using platinum metal (Pt) as a hydrogen cocatalyst and methanol as a sacrificial agent under UV-light irradiation. The energy conversion efficiency (defined as the ratio between the energy of solar-produced hydrogen and the energy of the incident sunlight) was 24% for black TiO_2_, which significantly exceeded that of typical TiO_2_-based materials (ca. 2%). The enhanced photocurrent in the UV range was attributed to an increase in donor density due to oxygen vacancies formed upon hydrogenation of TiO_2_. The high electronic density will contribute to shift the Fermi level of TiO_2_ toward the conduction band, enhancing charge separation and transport with an optimum concentration of oxygen vacancies. The authors used the original sample before hydrogenation as a blank sample (*Control Experiments and Blank Samples*); other blank samples, such as those obtained under similar annealing posttreatment under air or inert (argon-flow) atmosphere, would have been appropriate.

The original work (Chen et al., 2011) included photodegradation studies using methylene blue (MB) under irradiation conditions in the light-absorption range of the dye, and therefore, they will not be included in this discussion (*Dye-Sensitized Processes*). Among the few examples excluding dye-sensitized processes (Chen et al., 2015), some studies have reported on the visible-light activity of hydrogenated black TiO_2_ nanotubes during photodegradation of organic compounds (Danon et al., 2012; Zheng et al., 2012). Unfortunately, these claims could not be raised unambiguously based on the limited control experiments and reference samples provided (*Control Experiments and Blank Samples*). For instance, in one case, the TiO_2_ nanotubes collapsed into white TiO_2_ nanorods after the relevant annealing treatment (Zheng et al., 2012). Beyond the significant difference in surface area between these samples, the white nanorods were still significantly active (68%) under visible light points at considerable leaking of high-energy photons through the cutoff filter. Interestingly, cases of reactivity in the dark at room temperature have also been reported (Zeng et al., 2014), and hence, any conclusions regarding the photocatalytic behavior of black TiO_2_ should include outputs after testing during dark conditions (*Dye-Sensitized Processes*). The drop in photocatalytic activity of black TiO_2_ under visible-light irradiation has been widely observed in the literature (Chen et al., 2011; Hu, 2012). Incident-photon-to-current-conversion efficiency (IPCE) analysis (Figure 5B) (*Probing Visible Light Activity: Action Spectrum*) showed a decrease in activity from 95% in the UV range (370 nm) to ca. 1% in the visible range (>420 nm) (Wang et al., 2011; Hu, 2012). Fast charge carrier recombination of electron–hole pairs at trap states below the oxygen vacancies has been attributed as responsible for this decrease in activity in the visible range (Wang et al., 2017). It is rather surprising, however, to find claims of visible light activity during photodegradation of organic compounds using black TiO_2_.

Cycling experiments (Figure 5C) (*Material Stability*) confirmed that the sample was not merely a reservoir of hydrogen, and the amounts of hydrogen detected significantly exceeded that contained in the sample after hydrogenation. To the best of our knowledge, no further details have been provided about the long-term stability and performance of this material. The structure–property landscape of black TiO_2_ remains largely incomplete, and it will undoubtedly be subject of further active research throughout this new decade. Yet, following the photocatalytic work reported so far, this promising material will remain at the bottom of the qualitative pyramid shown in Figure 1.

## Conclusions

The area of photocatalysis may have reached maturity from a fundamental perspective (Ohtani, 2017), but the research efforts in photocatalyst design and engineering are still in their infancy. Undoubtedly, our understanding over the interplay of material properties and photocatalytic behavior will grow further in the new decade, taking advantage from in-depth characterization of materials under *operando* conditions and from closer synergy between theoretical and experimental studies (Tong et al., 2012). The intense research in material discovery, however, may be a bottleneck for the incorporation of promising photocatalysts into the market unless qualitative measures are considered.

Despite fruitful research in photocatalytic materials (Tong et al., 2012), overall quantum efficiencies remain low for widespread practical applications. A great deal of efforts have been particularly focused on hydrogen evolution. Yet, current solar-to-hydrogen conversion efficiencies are still below 1% and far from the 10% target for industrial use (Chen et al., 2017). As some authors have pointed (Chen et al., 2017), achieving this target will require apparent quantum yields of 40–60% at the low-energy side of the visible range (λ ≈ 600–700 nm), based on one-step photoexcitation processes. Nevertheless, the experimental evidence reported so far may call for alternative routes. The emerging work on plasmonic photocatalysts and the fine tuning of optical properties using quantum dots is particularly attractive in this sense (Linic et al., 2011; Boerigter et al., 2016). With qualitative measures in place, heterojunction and Z-scheme materials (Ran et al., 2014; Zhou et al., 2014; Li et al., 2016a) favoring electronic synergy, light absorption, and charge transport processes are still among the most promising routes toward photocatalyst commercialization. In contrast, the scalability options of rather complex systems—such as those obtained from defect engineering (Bai et al., 2018)—seem too convoluted for practical use, even when strategies toward their rational design were considered. The observation of the qualitative approaches as suggested in this perspective will allow for further strategies involving surface/interface chemistry, chemisorption properties, morphology control, selectivity, engineering of electronic structures to achieve overpotentials of redox reactions, and increasing photostability. In this sense, it is also crucial to encourage critical reviews (Minella et al., 2017; Rajaraman et al., 2020) to monitor and evaluate the current trends of photocatalytic materials in the literature.

## Author Contributions

The article was developed and written by RQ-C with scientific input and further discussion by IP. All authors contributed to the article and approved the submitted version.

## Conflict of Interest

The authors declare that the research was conducted in the absence of any commercial or financial relationships that could be construed as a potential conflict of interest.
